# A Diffusion Tensor Imaging Study: Relation of Wisconsin Card Sorting Covariates to White Matter Abnormalities in Traumatic Brain Injury

**DOI:** 10.3390/life15101633

**Published:** 2025-10-20

**Authors:** Aditya Prashant Kamble, Angel Liu, Dean Choi, Joseph Wu

**Affiliations:** 1 Charlie Dunlop School of Biological Sciences, University of California, Irvine, CA 92697, USA; 2 School of Social Sciences, University of California, Irvine, CA 92697, USA; angel.liu@cshs.org (A.L.); deanjc@uci.edu (D.C.); 3 School of Medicine, Department of Psychiatry, University of California, Irvine, CA 92697, USA; jcwu@hs.uci.edu

**Keywords:** Wisconsin Card Sorting Test, diffusion tensor imaging, traumatic brain injury, executive function, fractional anisotropy

## Abstract

New brain imaging modalities and neuropsychological testing tools are used to study neuronal changes in brain injuries such as mild traumatic brain injury (mTBI). Here we utilized diffusion tensor imaging (DTI) parameters and Wisconsin Card Sorting Test (WCST) variables to investigate patients with chronic mTBI. Neuropsychological assessments for mTBI evaluate impairments across a broad spectrum of executive functions. Our study aims to examine the relationship between fractional anisotropy (FA) and WCST covariates in patients with chronic mTBI. We hypothesize that patients who suffered chronic mTBI have significantly reduced FA in frontal white matter regions in association with significant deviation from standard percentile scores in WSCT. Utilizing multi-linear regression models alongside analyzing DTI scans, WCST covariates were linearly regressed to produce positive and negative contrasts to identify specific regions of interest (ROIs) with reduced FA. Results show that WCST covariates (such as percentile perseverative responses (Ep), non-perseverative responses (Enp), and conceptual response (CResp)) significantly deviate beyond standard percentile scores and correlate with lower FA in white matter regions in the frontal cortex, demonstrating executive function deficits. These frontal regions include the inferior frontal, superior frontal, and corpus callosum (CC), correlated with greater errors in WCST percentile scores. This study investigates the correlation between WCST covariates and DTI parameters as valuable tools in the diagnosis and prognosis of persistent cognitive impairment for patients with a history of chronic traumatic brain injury.

## 1. Introduction

Traumatic brain injury (TBI) is an alteration in normal brain function or any other evidence of brain pathology caused by an impact from external mechanical forces, such as a bump or jolt to the head, rapid acceleration or deceleration (i.e., fatal fall or vehicle accident), or penetration by a projectile (i.e., gunshot wound) [1]. The latest statistics from the Centers for Disease Control and Prevention (CDC) showed around 61,000 TBI-related deaths in the United States in 2019, averaging 166 TBI-related deaths every day [2]. The classification system for TBI—ranging from mild, moderate, to severe—is dependent on factors such as severity, amount of time loss of consciousness occurred for, possible post-traumatic amnesia, and evidence of brain damage in neuroradiological screening. While this system is highly consistent and viable for initial diagnosis, its utility as a long-term prognostic tool for detecting neuropsychological outcomes in mTBI is limited and rarely considers premorbid factors, underlying structural damage, and the impact of non-neurological factors beyond acute symptoms [3,4]. Though other regions may be involved, damage to the prefrontal cortex is strongly linked to executive dysfunction, and its effects on the quality of life in mTBI patients can range from unawareness of executive dysfunction to intrusive and crippling symptoms that negatively affect daily activities, emotional well-being, and social interactions.

Neuropsychological assessments administered during inpatient hospitalization or within clinical and research settings aid in the indication of new and/or existing neurological complications. Among various tests used to assess executive function, the most widely used is the Wisconsin Card Sorting Test (WCST). The WCST measures attention, working memory, concept formation, and set-shifting; parameters sensitive to cognitive processes including cognitive flexibility, problem-solving, and response maintenance [5,6,7]. Due to the internal structure of the test, most analyses include no more than two or three percentile scores (such as percentile perseverative responses (Ep), non-perseverative responses (Enp), and conceptual response (CResp)) to report a subject’s performance.

In conjunction with the WCST, the utility of diffusion tensor imaging (DTI) is a sensitive noninvasive imaging modality that evaluates the pathophysiology of head trauma through white matter nerve fibers. DTI can detect microstructural white matter disruptions in early post-injury, where conventional neuroradiological imaging is often not sensitive enough to detect persisting white matter injury [8]. DTI quantifies the microstructure of white matter by measuring the directionality of the molecular diffusion of water and allows for the 3D reconstruction of white matter fibers. The property of molecular diffusion in white matter tracts is defined as “anisotropic diffusion” and is measured as a parameter called fractional anisotropy (FA), a measurement of the direction consistency corresponding to the local loss of structural integrity. In mTBI, reduced global FA values have been seen in the corpus callosum (CC) and internal and external capsules in both acute and chronic stages of axonal injury [8].

In this study, we investigate the relationship between specific WCST covariates and white matter abnormalities in patients with chronic mTBI using DTI. We hypothesize that patients with poor WCST percentile scores (such as percentile Ep, Enp, and CResp) are associated with reduced FA in frontal white matter regions linked to persistent executive deficits following mTBI.

## 2. Materials and Methods

Fifteen chronic mTBI participants, 11 men and 4 women (mean age: 30.54 ± 11.37 years; mean education: 13.26 ± 1.62) at least 6 months post-injury were included with the following criteria(s): (a) Glasgow Coma Scale (GCS) score either at the emergency room (ER) or upon hospital admission of less than or equal to 13, (b) administration of the WCST, (c) underwent head MRI including DTI, and (d) no past history of severe neuropsychiatric conditions. Patients had no other significant neurological injury between their TBI evaluation, date of MRI scan, and neuropsychological testing.

Neuropsychological testing including the WSCT was administered to assess executive function. WCST Computer Version 4 was used. The computerized WCST presents 4 key cards and 128 response cards with geometric figures (i.e., square, triangle, circle, and star) varying in color, form, or number. The test starts by having the digitalized software present a stimulus card presented one at a time, and in turn, the interviewee must select a card matching the stimulus according to a criterion. After each turn, the interviewers tell the interviewee if their answer is correct or not, followed by learning through trial and error. After the interviewee yields ten consecutive correct matches, the classification criterion shifts without warning, demanding flexible working memory and concept formation to concurrently store and utilize information from the completed trials while processing the new cards presented. Once completed, the total and percentage of the following scores are observed: number of errors, perseverative errors, conceptual level responses, and non-perseverative errors.

All participants underwent MRI consisting of DTI and a high-resolution T1-weighted brain scan. MR imaging studies were performed on 3.0 T systems manufactured by Siemens or Philips Medical Systems. All images were processed for spatial normalization and smoothing using statistical parametric mapping software (SPM8 Version 6313)). DTI images were normalized using the Functional Magnetic Resonance Imaging of the Brain Structure Software (FSL).

Contrasts were created via a multiple regression model via SPM8 on MatLab (Version 7.1.0.246). Fifteen patients’ DICOMs were used alongside patient WCST percentile scores. Percentile scores were adjusted as covariates centered with an overall mean and intercept. Positive and negative transaxial Z-maps were generated by loading the mean image and positive and negative contrasts into Vinci (Version 2.54.0). For additional information on contrast and Z-Map generation, please refer to Appendix A.1 and Appendix A.2.

Regions of interest were obtained from the positive and negative transaxial Z-maps. Mean and standard deviation were then extracted from the regions of interest and saved to a spreadsheet for comparison. Various ROIs were drawn and included in a voxel-by-voxel multi-linear regression *t*-test by loading a mean image with contrast overlays into VINCI to be compared with 15 patient DTI image scans. ROIs also manually captured specific structures throughout the brain using contrasts and anatomically identified them via a 3-D map on Talairach Client (Version 2.4.3) along with assigned Talairach Atlas labels for an ROI’s given x, y, z coordinate. For additional information on ROI selection and Talairach, please refer to Appendix A.3.

IBM Statistical Package for Social Sciences (IBM SPSS Statistics 29.0.0.0) was used to run a single-sample *t*-test to compare patient WCST percentile scores to standard percentile scores and allowed for normalization. Multiple linear regression analysis was performed to examine the relationship of WSCT percentile covariates on the results of a neuropsychological assessment in brain regions with reduced FA (i.e., CC, corona radiata, frontal white matter, occipital white matter). Probability values of *p* < 0.05 were considered significant. Regression graphs and data visualization were created in SPSS and Excel using scatterplots with respective trend lines. Linear regression data were analyzed and interpreted using the established WCST percentile score protocol. All linear regression data were then compiled together to produce a comprehensive facet scatterplot using R studio (Version 2024.12.0, Build 467).

## 3. Results

### 3.1. Performance on WCST

#### 3.1.1. WCST Performance Mean Percentile Scores

Regional areas with FA were correlated with Ep, Enp percentile scores, and CResp via the SPM algorithm described above. Significant differences were observed on Ep (*p*  =  0.006), Enp (*p*  = 0.0075), and CResp (*p* = 0.0045) compared to standard control percentile scores on any of the examined covariates (*p*  >  0.05). WCST percentile scores increase as performance improves. Standard normal controls tend to have higher WCST percentile scores on average, whereas chronic mTBI patients tend to have lower WCST percentile scores on average (see Table 1).

#### 3.1.2. Variance Analysis

**Figure 1:**
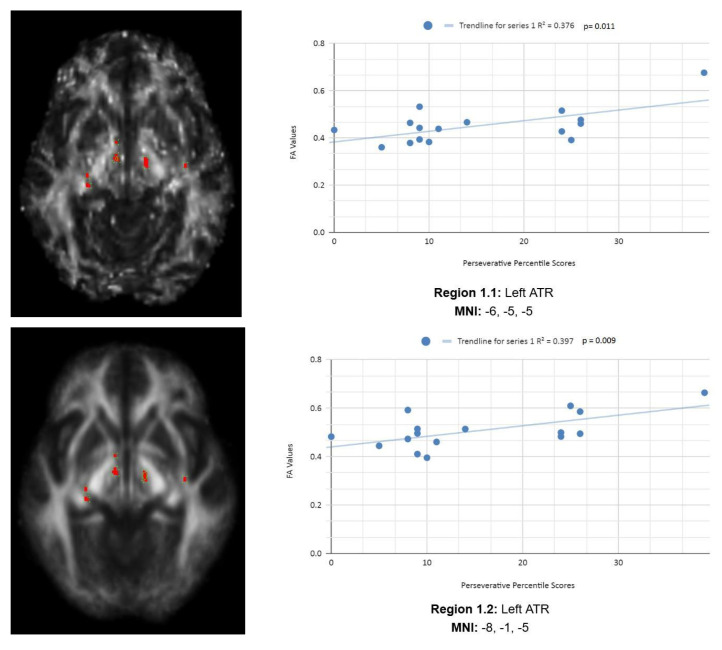

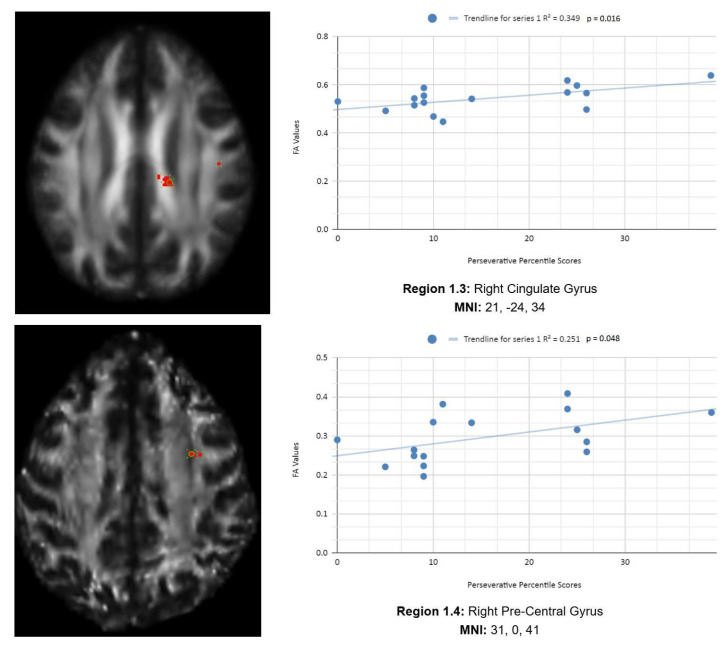
Perseverative Response Regions of Interest.

Variance analysis indicated significant group differences across the Ep, Enp, and CResp categories. We observed a statistically significant contrast between the mean WCST perseverative percentile score of the chronic mTBI patient subjects of 15.27 ± 6.03 compared to the mean standard control score of 87.6 ± 9.85. There was a statistically significant positive linear relationship between reduced percentile scores for perseverative error and reduced FA in the left anterior thalamic radiation (ATR), right cingulate gyrus, and right superior longitudinal fasciculus (SLF) regions (See Figure 1). No statistically significant negative correlations were observed in the Ep category.

**Figure 2:**
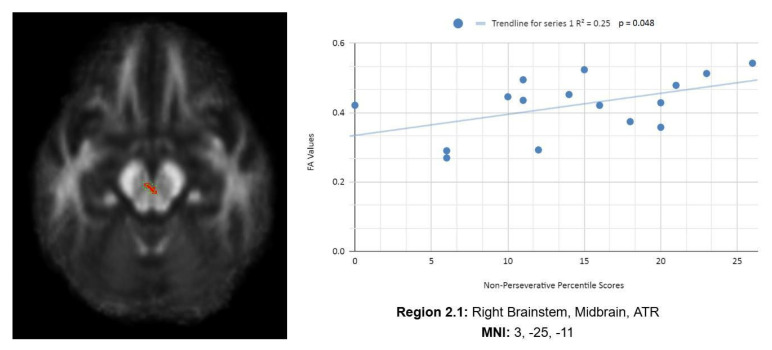

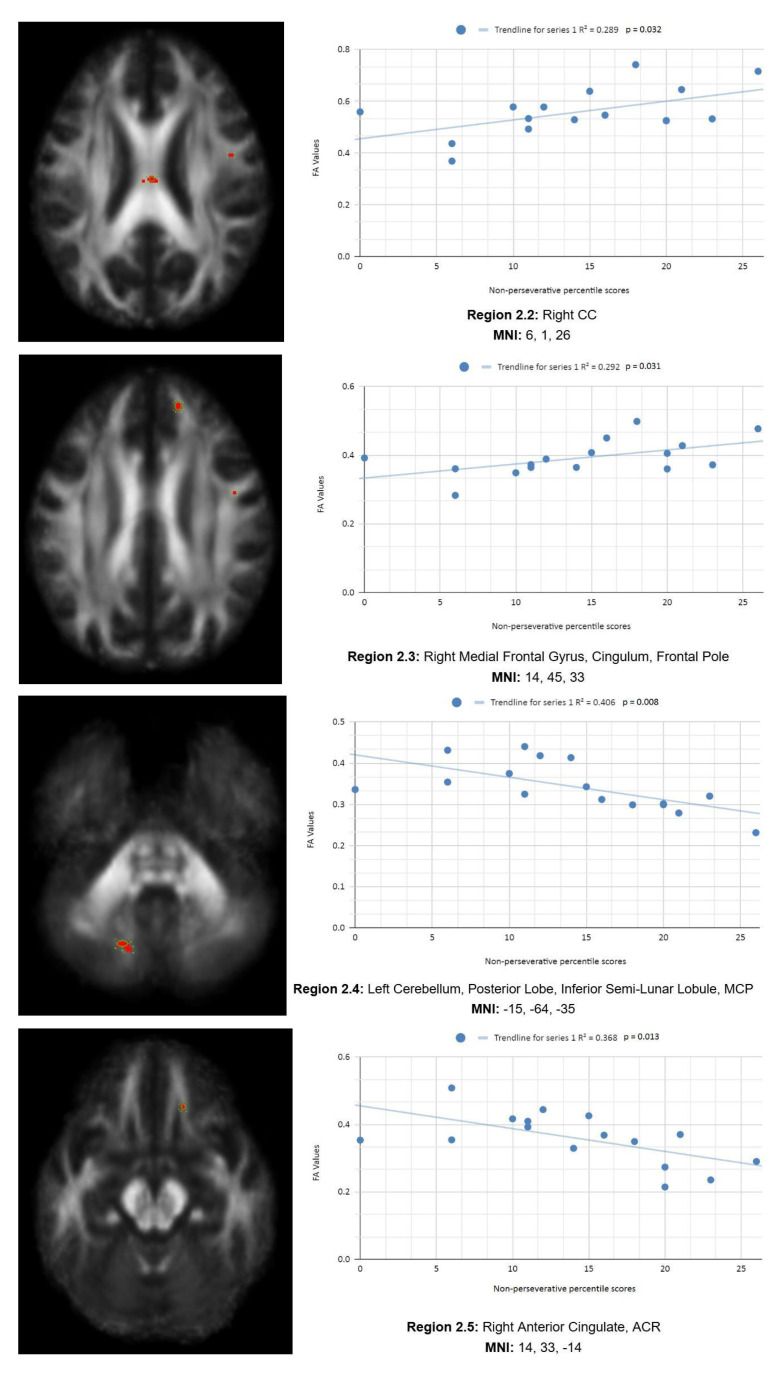

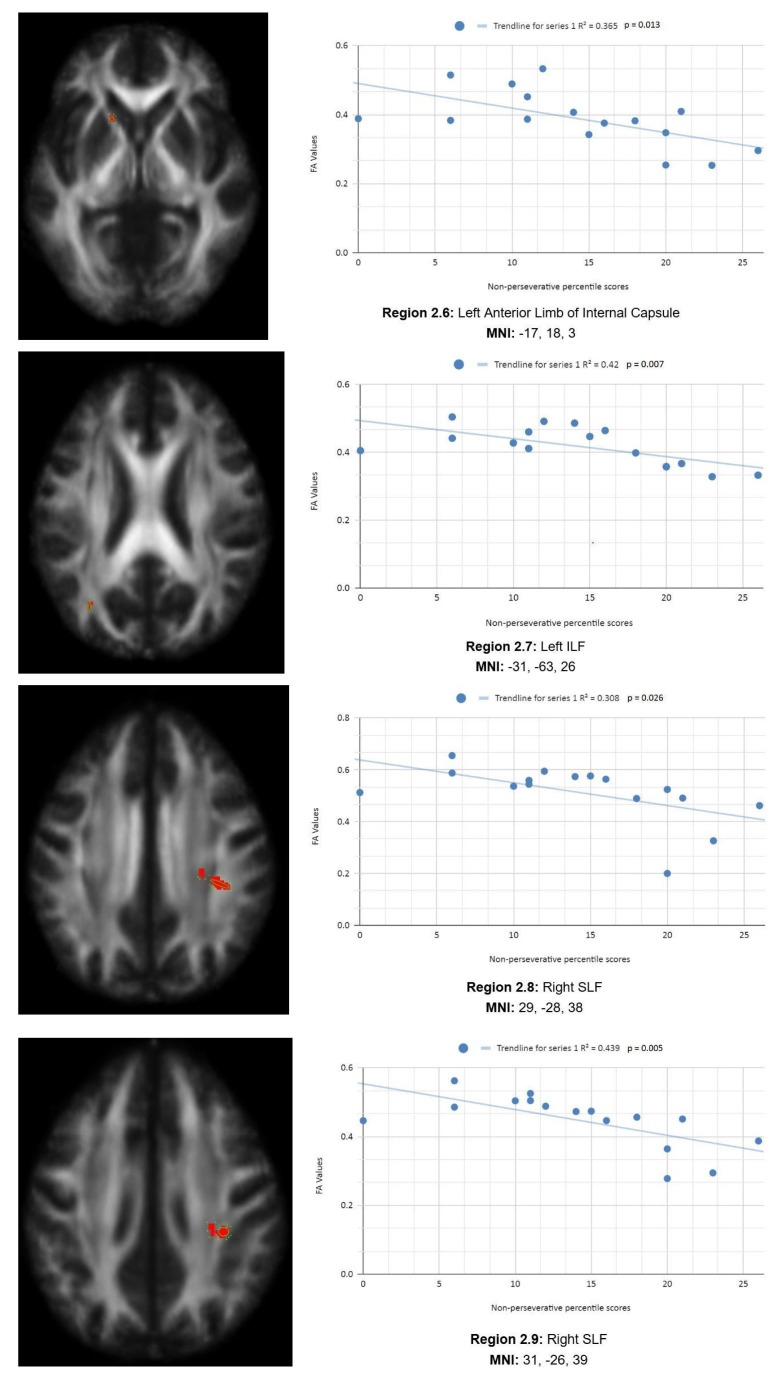
Enp linear regression graphs.

In addition, we also observed a statistically significant difference between the mean chronic mTBI patient WCST non-perseverative score of 16.67 ± 9.98 and the mean standard control non-perseverative error score of 93.46 ± 15.02.A statistically significant positive linear correlation can be observed between reduced non-perseverative percentile scores and reduced FA in the right CC, right ATR, and right frontal pole (see Figure 2). Statistically significant negative linear correlations can be observed for the following regions: middle cerebellar peduncle (MCP), anterior corona radiata (ACR), anterior limb of the internal capsule, left inferior longitudinal fasciculus (ILF), and right SLF (see Figure 2).

**Figure 3:**
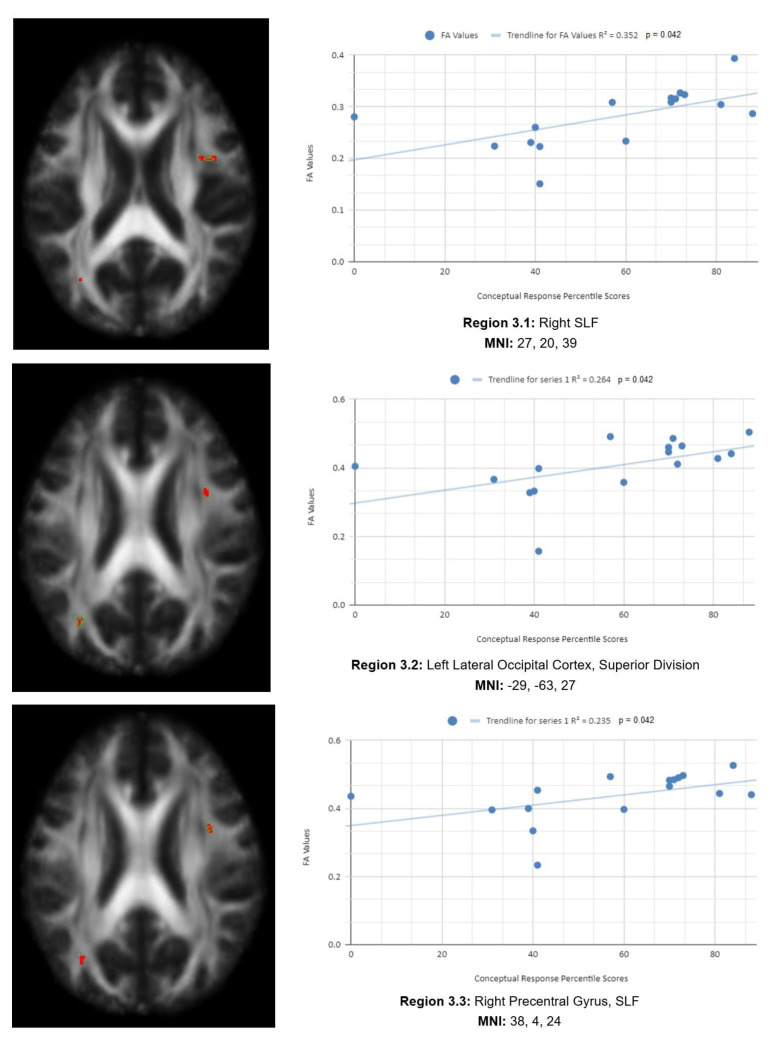

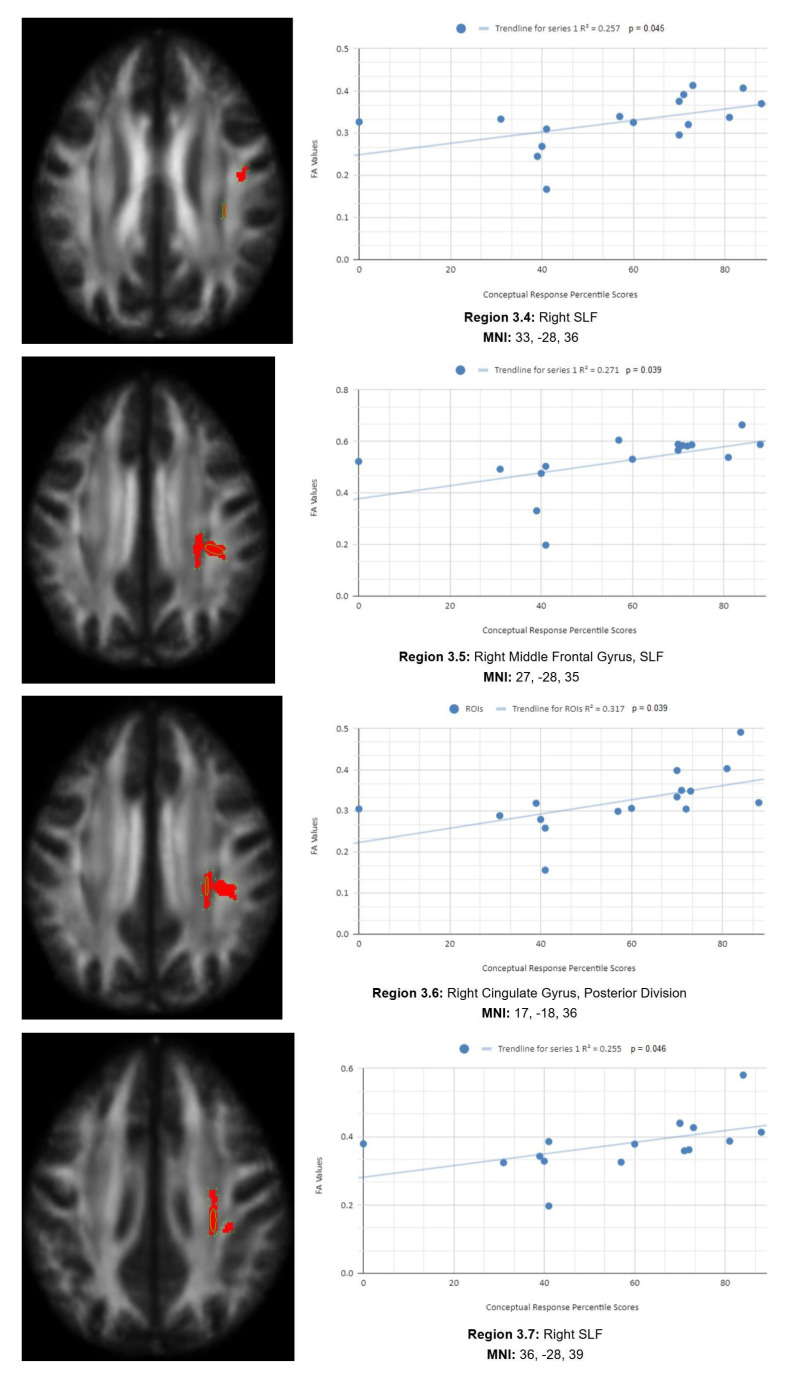

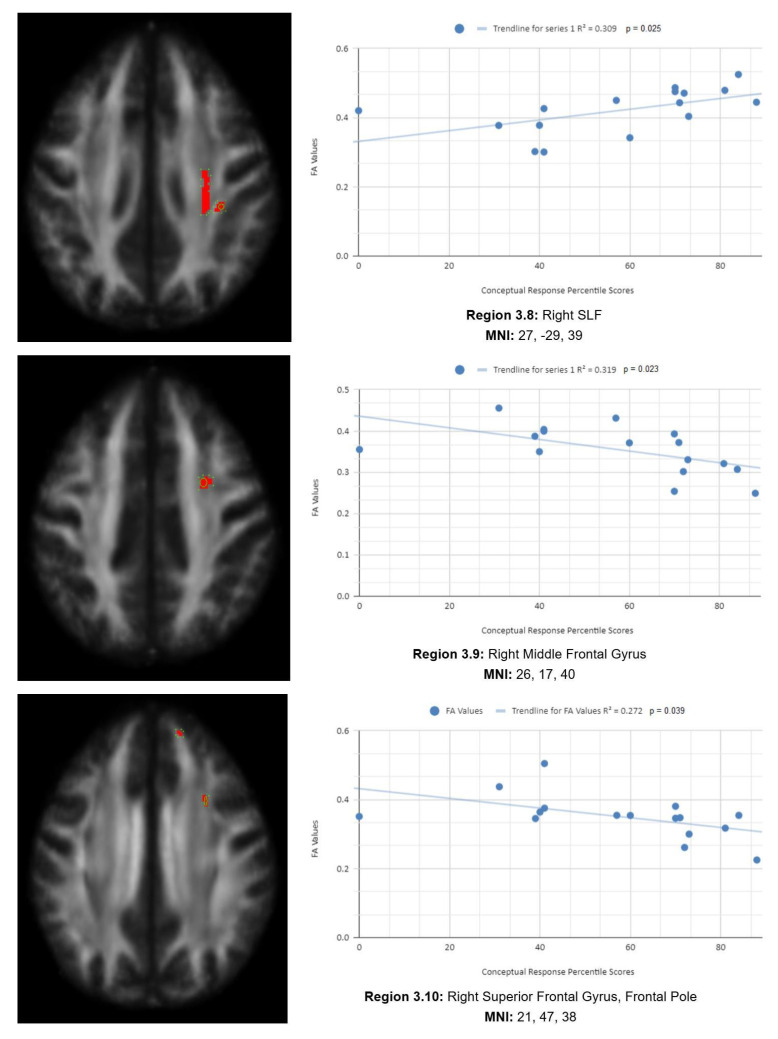
CResp regions of interest.

There is also a statistical difference between the mean chronic mTBI patient WCST CResp score (61.2 ± 18.57) and the normal mean standard WCST CResp score (89.73 ± 11.96). We also observed a statistically significant positive linear relationship between reduced CResp percentile scores and reduced FA in the right SLF, left lateral occipital cortex, and right cingulate gyrus (posterior division) regions (See Figure 3). Two statistically significant negative linear correlations were observed for CResp in the right middle frontal gyrus and right superior frontal gyrus, the frontal pole regions (see Figure 3).

### 3.2. DTI FA Analysis

#### 3.2.1. Ep

Figure 1 shows transaxial images of key brain regions of interest, including the ATR, cingulate gyrus, and precentral gyrus. It also shows FA values of each region correlated with Ep percentile scores through linear regression plots (Region 1.1, R^2^ = 0.376, *p* = 0.011; Region 1.2, R^2^ = 0.397, *p* = 0.009; Region 1.3, R^2^ = 0.349, *p* = 0.016; Region 1.4, R^2^ = 0.251, *p* = 0.048).

In the *X*-axis, perseverative percentile scores are depicted. In the *Y*-axis, FA values are depicted. Reduced FA values are consistent indicators of TBI, and reduced perseverative percentile scores are associated with higher errors and poorer performance, which are associated with cognitive impairment. A consistent positive association in four out of four correlations in this category validates the premise of the hypothesis that a decrease in FA results in poorer performance on the WCST.

#### 3.2.2. Enp

Figure 2 shows transaxial images of key brain regions of interest, including ATR, CC, and the frontal pole. These regions are correlated with reduced FA values and reduced Enp percentile scores in Excel to produce a scatterplot with the depicted correlations above (Region 2.1, R^2^ = 0.25, *p* = 0.048; Region 2.2, R^2^ = 0.289, *p* = 0.032; Region 2.3, R^2^ = 0.292, *p* = 0.031; Region 2.4, R^2^ = 0.406, *p* = 0.008; Region 2.5, R^2^ = 0.368, *p* = 0.013; Region 2.6, R^2^ = 0.365, *p* = 0.013, Region 2.7, R^2^ = 0.42, *p* = 0.007; Region 2.8, R^2^ = 0.308, *p* = 0.026; Region 2.9, R^2^ = 0.439, *p* = 0.005).

Reduced FA values are consistent indicators of mTBI, and reduced non-perseverative percentile scores are associated with higher errors and poorer performance, which are associated with cognitive impairment.

Enp percentile scores depict both positive and negative correlations, with three out of nine correlations being positive correlations. The positive correlations are observed in the ATR, CC, and frontal pole. The negative correlations are observed in the ACR, SLF, ILF, and left anterior limb of the internal capsule. The *X*-axis depicts the non-perseverative percentile scores (0–100 percentile) in each graph. The *Y*-axis displays FA values (0–1).

#### 3.2.3. CResp

Figure 3 shows transaxial images of key brain regions of interest, including the right SLF, right cingulate gyrus, and left lateral occipital cortex, superior division. These regions are correlated with reduced FA values and reduced CResp percentile scores using Excel (Region 3.1, R^2^ = 0.352, *p* = 0.042; Region 3.2, R^2^ = 0.264, *p* = 0.042; Region 3.3, R^2^ = 0.235, *p* = 0.042; Region 3.4, R^2^ = 0.257, *p* = 0.045; Region 3.5, R^2^ = 0.271, *p* = 0.039; Region 3.6, R^2^ = 0.317, *p* = 0.039; Region 3.7, R^2^ = 0.255, *p* = 0.046; Region 3.8, R^2^ = 0.309, *p* = 0.025; Region 3.9, R^2^ = 0.319, *p* = 0.023; Region 3.10, R^2^ = 0.272, *p* = 0.039).

In the *X*-axis, CResp percentile scores are observed (0–100 percentile). In the *Y*-axis, FA values are observed (0–1). Reduced FA values are consistent indicators of TBI, and reduced conceptual percentile scores are associated with higher errors and poorer performance, which are associated with cognitive impairment. A relatively consistent positive association, with 8 out of 10 correlations being positive in this category, supports the premise of the hypothesis that a decrease in FA results in poorer performance on the WCST.

#### 3.2.4. Analysis of Multi-Linear Regression

**Figure 4:**
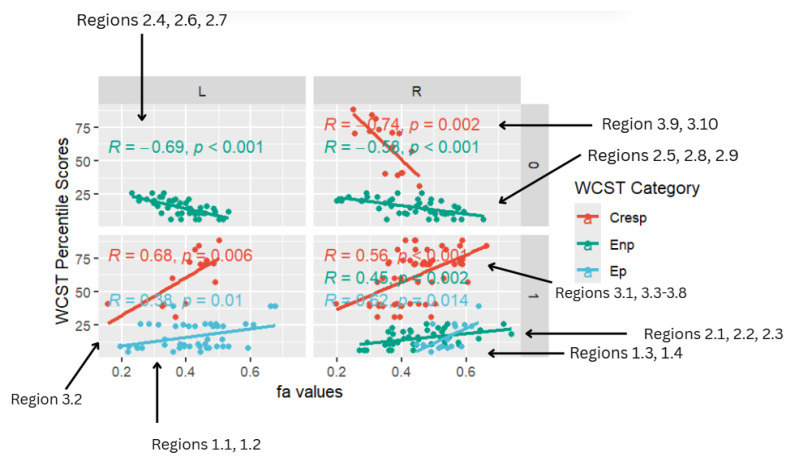
Facet scatterplot.

Multi-linear regression analysis was performed to collectively examine regions showing a significant reduction in FA in participants with the corresponding WCST covariates (Ep, Enp, and CResp). Contrast and lesion-related adjustments were made in partial multi-linear regression analyses, revealing significant connections between WCST covariates and specific left (L) and right (R) frontal–temporal and frontal–posterior white matter regions with reduced FA. Important correlates of reduced WCST percentile scores with probability values of *p* < 0.05 were considered statistically significant. These statistically significant regional correlations are displayed in Figure 4.

The multi-linear regression analysis of the relationship between WSCT covariates and brain regions with abnormal FA values focused on the CC, frontal white matter, and occipital white matter. A statistically significant positive linear relationship was observed between reduced WSCT (Ep, Enp, CResp) percentile scores across all three WCST categories and reduced FA in frontotemporal (CC, frontal pole, SLF, ATR, and middle frontal gyrus), temporal (lateral occipital cortex, ILF), limbic (ACR), and fronto-posterior (MCP) regions.

Notably, there were positive correlations with the right precentral gyrus and cingulate gyrus FA (r = 0.62, *p* = 0.014) and left ATR FA (r = 0.38, *p* = 0.01) for Ep, the right brainstem, right CC, and right medial frontal (r = 0.45, *p* < 0.002) for Enp, and left lateral occipital cortex (r = 0.68, *p* = 0.006), right frontal SLF, and right cingulate gyrus (r = 0.56, *p* < 0.001) for CResp.

Overall, the multi-linear regression analysis largely found statistically significant positive correlations across all WCST categories (Ep, Enp, CResp), with a large majority of correlations displaying a positive trend.

Figure 4 is a facet scatterplot produced in R Studio. It compiles all the statistically significant data points and correlations into one medium and summarizes the entire multi-linear regression analysis. Panels on the left side show regions in the left (L) frontal–temporal and frontal–posterior white matter regions, while panels on the right-side show regions in the right (R) frontal–temporal and frontal–posterior regions. The topmost panels show regions mapped using negative z-maps (0) and the bottom panels show regions mapped using positive z-maps (1). In each panel, WCST percentile scores from each WCST covariate (CResp, Ep, and Enp) are displayed in correlation with FA values at a specific brain region (L/R) that was mapped out from a specific kind of z-map (0/1).

Five out of eight of the correlations observed in the facet scatterplot were positive associations between WCST percentile scores and FA values. Positive correlations show that WCST percentile scores decrease across all WCST categories where FA values also decrease. Lower WCST percentile scores indicate poorer performance on the test in association with reduced executive function.

In the Ep category, two statistically significant positive correlations are displayed between perseverative percentile scores and FA values in the left and right frontotemporal and frontal–posterior white matter regions derived from positive z-maps. These regions include Regions 1.1 (Left ATR) and 1.2 (Left ATR) on the bottom left panel shown above. Regions 1.3 (right cingulate gyrus) and 1.4 (right precentral gyrus) are shown on the bottom right panel. The positive correlations in these specific regions suggest that as perseverative percentile scores decrease, FA values also decrease. DTI FA data are validated by WCST perseverative percentile score data in the ATR, cingulate gyrus, and precentral gyrus.

One overall positive correlation was also observed between non-perseverative (Enp) percentile scores and FA values in the left and right frontotemporal and frontal–posterior regions derived from negative Z-maps (0). These regions include Regions 2.1 (Right Brainstem, Midbrain, ATR), 2.2 (Right CC), and 2.3 (Right Medial Frontal Gyrus, Cingulum, Frontal Pole), which were correlated together in the bottom right panel.This overall positive correlation means that as non-perseverative percentile scores decrease, so do FA values. DTI FA data are validated by WCST non-perseverative percentile scores.

Two positive correlations were also observed between CResp percentile scores and FA values in the left and right frontotemporal and frontal–posterior regions derived from positive z-maps (1). The correlation of Region 3.2 (left lateral occipital cortex, superior division) is depicted on the bottom left panel. In the bottom right panel, regions 3.1 (right SLF), 3.3 (right precentral gyrus, SLF), 3.4 (right SLF), 3.5 (right middle frontal gyrus, SLF), 3.6 (right cingulate gyrus, posterior division), 3.7 (right SLF), and 3.8 (right SLF) are correlated together with CResp percentile scores. Positive correlations in this category mean that as CResp percentile scores decrease, so do FA values. DTI FA data are validated by WCST CResp percentile scores.

## 4. Discussion

This study investigates the relationship between executive dysfunction and white matter microstructure in chronic mTBI patients using DTI and WSCT covariates. Our findings showed that poor WCST percentile scores in the perseverative, conceptual, and non-perseverative categories are associated with impaired executive function in multiple frontal white matter regions. Anatomically, given their site at the front of the brain and their large size, the frontal lobes are particularly vulnerable to TBI, with memory, attention, and cognitive shifting functions being the most frequently affected domains in mTBI [9,10,11]. Reductions in total brain volume and cerebral atrophy are common and well-established outcomes of TBI. Previous publications have analyzed subtle volumetric changes to predict clinical outcomes post-TBI based on segmentation techniques available in Free Surfer and concluded that regional morphometrics were correlated with deficits in cognitive outcomes with volumes of gray matter structures being strongly associated with chronic damage to related white matter tracts and less associated with measures of white matter integrity in acute scans [12,13]. Main findings of our study show that a positive correlation was observed consistently between reduced perseverative error percentile scores and reduced FA values in four key frontal white matter regions, such as the left SLF (r = 0.38, *p* = 0.01), along with the cingulate gyrus and ATR (r = 0.62, *p* = 0.014).

Patients with mTBI typically scored in lower percentiles compared with the national average and had multiple white matter regions with reduced FA in their brain scans, predominantly involving cerebral white matter, frontal pole, and CC. There was no significant relation between the time interval after injury and our DTI and fiber tracking findings. Reduced FA values were associated with structural damage and mTBI. Higher FA was also noted in some reports as being present in chronic TBI patient populations; however, it is less common and is usually associated with chronic inflammation or gliosis [14,15]. Linear regression data with a positive correlation was interpreted as an association between FA and WCST percentile scores; as FA values reduced, so did WCST percentile scores.

To measure executive function using the WCST, it is crucial to understand that greater dysfunction results in higher scores in perseverative errors, and lower percentile scores. Ep involves participants matching a card using the same rule (i.e., color, form, or number) used in the immediate previous match, regardless of whether the response is correct or not. Perseverative errors suggest an incapacity to inhibit an automatic response despite knowing from feedback that the response is incorrect [7,12,16]. This suggests that patients who have reduced perseverative percentile scores, indicating cognitive inflexibility, have reduced white matter integrity, which is critical for maintaining cognitive flexibility and executive control. 

For conceptual percentile scores, there was a positive correlation with lower FA values in the frontal lobe, specifically in the SLF for patients with lower CResp percentile scores. Positive correlations observed in the category response support the premise of our hypothesis that lower FA is associated with lower WCST percentile scores, poorer performance, and higher errors. For the category response specifically, this correlation indicates that patients with lower scores have difficulties in forming concepts and having insights, even with flexible answers. The SLF connects frontal regions to parietal and occipital lobes, suggesting that reduced connectivity could contribute to difficulties in conceptual understanding [12,17]. This suggests selective neurodegeneration in significant regions. From previous studies, this could suggest that axonal myelination during maturation may be altered in mTBI, where we found reduced FA in patients with brain trauma that was not progressive [4,7]. 

For the Enp category, results showed both a negative and positive correlation in different regions of the brain. A negative correlation was observed in limbic, ILF, and sub-lobar regions, whereas a positive correlation in the CC, ATR, and frontal pole was observed. mTBI patients have lower FA in white matter tracts of the genus of the CC and midbrain. The significant correlation in Enp between reduced Enp percentile scores and reduced FA in the CC highlights the role of interhemispheric communication in detection of non-perseverative errors. Damage to the CC as indicated by reduced FA has been shown to impair coordination between hemispheres which may play a crucial role in detection of non-perseverative errors.

The negative correlations in the Enp and CResp categories contradict the premise of the hypothesis that FA reduction is associated with reduced non-perseverative percentile scores. These results indicate that higher FA is associated with poorer performance in the non-perseverative category. While most DTI studies associate low FA values with TBI, a handful of reports say the same for high FA [18,19]. The inconsistency in the literature regarding elevated versus reduced FA values in TBI may be explained by differences between acute and chronic phases of injury. Elevated FA has been observed primarily in acute cases, where cytotoxic edema may restrict extracellular diffusion and lead to increased directional water movement within axons [18,20]. In contrast, decreased FA is more commonly reported in chronic TBI and is often linked to structural alterations such as fiber misalignment, axonal degeneration, edema, or diffuse disruption of white matter architecture [12,17]. In addition, observed higher FA in chronic mTBI populations could also be due to possible chronic inflammation, gliosis, or axonal scarring that alters the pattern of water diffusion in white matter tracts [14,15].

The mechanism of this phenomenon is not completely understood yet. However, it may explain why abnormally high FA was observed in some regions in the Enp and CResp correlations. The regions that are positively correlated with non-perseverative percentile scores and FA values are involved in processing visual information and transmitting signals between the frontal and temporal lobes. For example, the CC has been known to facilitate communication between the two hemispheres of the brain, and its reduced integrity in chronic mTBI patients suggests that the interhemispheric transfer of information may be impaired, contributing to deficits in higher-order conceptual processing [4,21]. The reduction in FA in these tracts may reflect disruptions in the brain’s ability to integrate sensory information with higher-order cognitive processes, potentially explaining patients’ difficulties with non-perseverative errors, which require adaptability and learning.

## 5. Limitations

A few limitations of this study must be considered, starting with how we define regions with abnormally reduced FA. The interpretation of reduced FA as a marker of WM damage should be considered with caution as FA can be related to several tissue characteristics, such as axon density, axon diameter, degree of myelination, alignment of fibers within a voxel, and partial volume effects. We also did not examine the role of other key individual covariates, such as individual differences in emotional regulation that have been linked to attention control processes. Longitudinal and further studies are needed. Careful integration with neuropsychological measures of working memory, processing speed, and other cognitive functions will result in a better overall understanding of prognosis and long-term outcomes in TBI. We compared patients with a mean image in a voxel-based ROI z-score analysis. Since ROIs were performed manually, this may cause an overestimation of lesion size in these regions [11]. Categories Completed (CatComp) as a covariate did not lead to any statistically significant results as there were no comparable differences between patient mean percentile scores and standard mean percentile scores. In addition, FA differences for CatComp did not reach statistical significance due to relatively small statistical power and led to statistical skewing.

Although the WCST is a widely used and highly validated diagnostic tool for quantifying executive function, many issues remain complicated in both individual evaluation and group comparisons. Findings from other research have shown that performance on the WCST cannot be interpreted in isolation as an index of frontal lobe damage [16,22]. For example, the positive correlation between reduced Ep percentile scores and reduced FA in the frontal white matter regions like the SLF is consistent with prior findings that show that the frontal lobes are particularly vulnerable in TBI, and these regions are responsible for higher-order cognitive tasks, including decision-making and problem-solving [10,11]. In general, applying conservative cognitive malingering cutoffs to classify deficient effort can also fail to recognize TBI patients with more debilitating and complicated psychosocial symptoms. This can ultimately affect motivation and the validity of the WCST as a neuropsychological assessment tool. Several studies on mTBI [21,23,24] have demonstrated a reduction in white matter integrity in specific tracts, mostly in the forceps minor, major of the CC, and frontal–occipital fasciculus, in patients with “chronic” mTBI. Furthermore, these results add to coinciding evidence developing in support of the clinical utility of DTI in predicting post-injury outcomes [25,26]. Though chronic mTBI has been studied through DTI before, this study is one of the first to measure DTI validity directly through the lens of WCST covariates in this chronic mTBI patient population.

Our multiple linear regression analysis demonstrates that specific executive dysfunctions, as captured by WCST covariates, are associated with microstructural white matter damage in regions crucial for executive functioning and cognitive control. Patients may display this pattern because these measures are exclusive. Excessive perseverative errors lead to non-perseverative covariates being insignificant as there is nothing left to measure.

Predicting cognitive outcomes is an integral part of the early rehabilitation process, medical care, and experimental therapies that are aimed at improving long-term prognosis. Many publications have demonstrated the usefulness of WCST and DTI in detecting microstructural disruption in concussions. Previous studies [16,26,27] investigating the relation between executive dysfunction from neuropsychological testing and cerebral pathological changes in TBI showed significant deviation from standard percentile scores, providing a subtle indication of impairment in executive function in frontal regions of the brain. Regions of diffuse axonal injury that appeared normal with conventional neuroimaging but with DTI in the acute phase can show changes due to cerebral edema that can be reversible and therefore does not reflect the chronic brain changes related to cognitive impairment [28,29].

Our results are similar, where the positive correlation between reduced Ep percentile scores and reduced FA in the frontal white matter regions, like the SLF, is consistent with prior findings which show that the frontal lobes are particularly vulnerable in TBI. The purpose of this study was to test the hypothesis that patients with post-injury cognitive impairment have DTI-derived neuroimaging biomarkers specifically associated with poorer-performing WCST covariates. These regions are responsible for higher-order cognitive tasks, including decision-making and problem-solving; patients could experience a significant decrease in quality of life for patients in daily tasks post-injury [10,11].

Additional limitations allude to the fact that our study has only analyzed a handful of key regions in the brain, and the correlations observed between WCST covariates and FA values (though statistically significant) are weak associations that need further validation from additional correlations and analyses.

## 6. Conclusions

In conclusion, results from our study show that mTBI patients whose WCST covariate (e.g., percentile Ep, percentile Enp, percentile CResp) percentile scores significantly deviate beyond standard percentile scores show lower FA in white matter regions, mainly in the frontal cortex, including the SLF, ACR, frontal pole, and the CC, demonstrating executive function deficits. Our results are consistent with the previous literature, as WCST covariates are a considerably accurate measure in correlating reduced executive performance to brain regions and strongly support the hypothesis that WCST performance in mTBI patients is associated with specific patterns of white matter disruption.

There is a shortage of standardized techniques for detecting and predicting the potential long-term effects of traumatic brain injuries. mTBI is a clinical diagnosis due to the absence and limitation of validated diagnostic biomarkers. This study adds to the literature by confirming previous findings, including statistically significant relationships between validity indicators of neuropsychological pre-screenings (WCST covariates) and regional FA values to help detect TBI, as evidenced by their ability to somewhat effectively discriminate brain regions impacted by head injury or impact. The cause of FA reduction in brain white matter in TBI has not been fully understood. Chronic injuries can be difficult to recognize on imaging if they are small and peripherally located. However, decreases in FA are sensitive indicators of histological abnormality. Since FA values are lower in gray matter than in white matter and measurements are affected by noise in the gray matter area, the interpretation of FA reduction in gray matter is more difficult than in white matter. Precursor neuropsychological testing, specifically the relationships between WCST performance and DTI measures, allows for the identification of altered myelination as a possible source of FA reduction. Our correlation analysis revealed multiple statistically significant correlations between various total WCST covariate percentile scores and FA regions in white matter.

Our study findings aim to contribute to the understanding of how executive dysfunction after mTBI is linked to structural brain damage with DTI and WCST covariates. With mTBI, important implications for rehabilitation strategies post-injury should focus on the identification and rehabilitation of any lasting neurological difficulties from their injury. We have shown significant deviation of WCST percentile scores in specific covariates, indicating potential traumatic axonal injury in white matter as evidenced by a reduction in FA in white matter tracts as shown in DTI. WCST covariates correlate with DTI-derived scalar measures (FA), revealing WCST covariates as a valuable tool to identify executive dysfunction linked to changes in white matter integrity in frontal brain regions. Using multi-linear regression, results show multiple significant ROIs in correlation with WCST explanatory covariates, specifically Ep, Enp, and CResp. These covariates highlighted significantly lower FA values in white matter tracts in nine preference sites, mainly in the frontal lobe, posterior, temporal, sub-lobar, and limbic regions. In both clinical and research settings, DTI and WCST (among other imaging modalities and neuropsychological tests) prove to be valuable, reliable, and accessible tools to highlight a significant correlation between test performances and imaging biomarkers linked to chronic mTBI injuries.

## Figures and Tables

**Table 1:** WCST mean patient percentile scores compared to mean control percentile scores across Ep, CResp, and Enp.

WCST Category	Mean Patient Percentile Scores	Mean Control Percentile Scores
Ep	15.27 ± 6.03	87.6 ± 9.85
CResp	61.2 ± 18.57	89.73 ± 11.96
Enp	16.67 ± 9.98	93.46 ± 15.02

For each WCST category (Ep, CResp, and Enp), the mean patient percentile scores are significantly lower than standard control percentile scores. Lower percentile scores indicate poorer performance in each WCST category. Patients performed poorer than standard controls on average in the WCST.

## Data Availability

The original contributions presented in this study are included in the article. Further inquiries can be directed to the corresponding author.

## Abbreviations

The following abbreviations are used in this manuscript:

ROI	Region of Interest
FA	Fractional Anisotropy
WCST	Wisconsin Card Sorting Test
DTI	Diffusion Tensor Imaging
Ep	Perseverative Response
Enp	Non-Perseverative Response
Cresp	Conceptual Response
TBI	Traumatic Brain Injury
mTBI	Mild Traumatic Brain Injury
CC	Corpus Callosum
ATR	Anterior Thalamic Radiation
SLF	Superior Longitudinal Fasciculus
ILF	Inferior Longitudinal Fasciculus
MCP	Middle Cerebellar Peduncle
ACR	Anterior Corona Radiata

## Appendix A

### Appendix A.1

To produce contrast masks, relative threshold implicit masking was applied with a threshold of 0.8. Global normalization was set to be proportional with a grand mean scale value of 50. The global calculation was omitted. Estimated parameters generated SPM.mat contrast files where Positive (0 1) and Negative (0 −1) t-contrasts were created. Contrasts were adjusted with no applied masking, no *p*-value adjustments, threshold val = 0.01, and extent voxels = 30. Whole-brain contrast masks and figures were then generated and saved for ROI processing.

### Appendix A.2

To produce Z-Maps, mean images for the 15 chronic mTBI patients were generated using SPM8 on MatLab. All 15 patients’ DICOM NIFTI files were inputted into SPM8 “ImCalc” to generate mean images with the expression “mean (x)”. Images were read into the data matrix with no implicit zero masking, trilinear interpolation, and INT16-signed short data type.

### Appendix A.3

To manually draw ROIs, positive and negative contrasts were overlaid by the mean image to create Z-Maps with the following parameters X/Y: 5 × 4, Cross Marker: Uncheck Show, Skip Planes: 4, Range of Planes: first = 145, last = 50. Respective positive and negative prominent ROIs were then selected from Z-Maps for analysis with 15 patient NIFTI files loaded into Vinci and processed using the ROI 2D define tool. Coordinates taken from the SPM figure were plugged into the Talairach client to locate anatomical regions. Additional labels were also added from FslEyes if Talairach did not identify the region.

## Appendix B

To produce the facet scatterplot, data was first split categorically into five different columns in Excel (fa values, WCST Category, WCST Percentile Scores, Hemisphere, and Contrasts). The Excel sheet containing the data file was then imported into R studio and the following code was run:

library(ggplot2) library(ggsci) library(ggpubr) ggplot(Insert_Data_File_Name, aes(x = `fa values`, y = `WCST Percentile Scores`, color = `WCST Category`)) + geom_jitter(height = 0, width = 0) + facet_grid(Contrast ~ Hemisphere) + scale_color_npg() + geom_smooth(method = "lm", se = FALSE) + stat_cor(p.accuracy = 0.001, r.accuracy = 0.01, label.x.npc = "left", label.y.npc = "top")

Libraries in R Studio were imported and called (ggplot2, ggsci, ggpubr). In the ggplot function, the name of the Excel file was added to call the data in the file. The aesthetic (aes) function was used to define the main X-Y variables in the scatterplot (WCST percentile scores and FA values). The “geom_jitter” function determined the height and width of the plot, along with increments. The facet grid function helped establish the contrast and hemisphere columns in Excel as sections in the facet grid. The “scale_color_npg()” and “geom_smooth” functions defined additional aesthetics of the visual such as color categorization and best fit lines for each correlation. The “stat_cor” functions ran statistical correlations on the data points and displayed *p*-values and R-values for each relationship investigated.
